# The Role of Metabolic Factors in Renal Cancers

**DOI:** 10.3390/ijms21197246

**Published:** 2020-09-30

**Authors:** Jacek Rysz, Beata Franczyk, Janusz Ławiński, Robert Olszewski, Anna Gluba-Brzózka

**Affiliations:** 1 Department of Nephrology, Hypertension and Family Medicine, Medical University of Lodz, 90-549 Lodz, Poland; jacek.rysz@umed.lodz.pl (J.R.); bfranczyk-skora@wp.pl (B.F.); 2 Department of Urology, Institute of Medical Sciences, Medical College of Rzeszow University, 35-055 Rzeszow, Poland; janlaw@poczta.onet.pl; 3 Department of Gerontology, Public Health and Education, National Institute of Geriatrics Rheumatology and Rehabilitation, 02-106 Warsaw, Poland; robert.olszewski@me.com; 4 Department of Ultrasound, Institute of Fundamental Technological Research, Polish Academy of Sciences, 02-106 Warsaw, Poland

**Keywords:** renal cell carcinoma, obesity, insulin resistance, diabetes mellitus, lipid disorders

## Abstract

An increasing number of evidence indicates that metabolic factors may play an important role in the development and progression of certain types of cancers, including renal cell carcinoma (RCC). This tumour is the most common kidney cancer which accounts for approximately 3–5% of malignant tumours in adults. Numerous studies indicated that concomitant diseases, including diabetes mellitus (DM) and hypertension, as well as obesity, insulin resistance, and lipid disorders, may also influence the prognosis and cancer-specific overall survival. However, the results of studies concerning the impact of metabolic factors on RCC are controversial. It appears that obesity increases the risk of RCC development; however, it may be a favourable factor in terms of prognosis. Obesity is closely related to insulin resistance and the development of diabetes mellitus type 2 (DM2T) since the adipocytes in visceral tissue secrete substances responsible for insulin resistance, e.g., free fatty acids. Interactions between insulin and insulin-like growth factor (IGF) system appear to be of key importance in the development and progression of RCC; however, the exact role of insulin and IGFs in RCC pathophysiology remains elusive. Studies indicated that diabetes increased the risk of RCC, but it might not alter cancer-related survival. The risk associated with a lipid profile is most mysterious, as numerous studies provided conflicting results. Even though large studies unravelling pathomechanisms involved in cancer growth are required to finally establish the impact of metabolic factors on the development, progression, and prognosis of renal cancers, it seems that the monitoring of health conditions, such as diabetes, low body mass index (BMI), and lipid disorders is of high importance in clear-cell RCC.

## 1. Introduction

An increasing number of evidence indicates that metabolic factors may play an important role in the development and progression of certain types of cancers [1]. The presence of metabolic syndrome has been shown to be associated with greater risk of prostate, liver, pancreatic, bladder, colorectal, cervical, and postmenopausal breast cancers [2,3,4,5,6]. Moreover, the prognosis of patients with cancers and concomitant metabolic syndrome has been suggested to be worse. Obese patients with prostate cancer were shown to be more likely to develop high-grade and aggressive cancer [7]. The impact of metabolic factors has also been shown in patients with renal cell carcinoma. 

Renal cell carcinoma (RCC) is the most common kidney cancer which accounts for approximately 3–5% of malignant tumours in adults and its incidence is still rising as an effect of modern lifestyle and high prevalence of comorbidities [8]. According to WHO estimations, RCC is the 6th most frequently diagnosed malignancy in men, the 10th commonest tumour in women, and the 13th commonest cause of cancer death [9]. The highest incidence of renal cell carcinoma is observed in North America and Europe [10]. Owing to the development of imaging technology and better healthcare, the frequency of detection of early-stage renal tumours has increased; however, a considerable percentage of patients have metastases in the lungs, liver, brain, or lymph nodes at the time of diagnosis [11,12]. Renal cell carcinoma belongs to a heterogeneous group of primary kidney adenocarcinomas originating from renal parenchyma, which comprises three histologically different types: clear cell (80–90%), papillary (10–15%), and chromophobe (4–5%) [8]. The loss of von Hippel-Lindau (VHL) tumour suppressor accompanied by subsequent stabilization of hypoxia-inducible factor (HIF) responsible for extensive metabolic reprogramming is the most frequent cause of clear-cell renal cell carcinoma [13]. Obesity, smoking, and hypertension are among the lifestyle-associated risk factors of RCC present in up to 50% of cases [10,14,15]. Moreover, diabetes mellitus (DM) type 2 in women, high body mass index (BMI), and increased blood pressure in men, as well as elevated levels of triglycerides, were suggested to be independent RCC risk factors [16,17,18]. Numerous studies indicated that concomitant diseases, including DM and hypertension, as well as obesity, insulin resistance and lipid disorders, may also influence the prognosis and cancer-specific overall survival [19,20,21]. However, the results of studies concerning the impact of metabolic factors on RCC are conflicting. It remains unclear whether these factors alter the risk independently, or they interact to increase the risk of RCC. Irrespective of their manner of action, it seems that worse outcomes could be the result of the presence of metabolic factors per se.

## 2. Obesity

**Figure 1:**
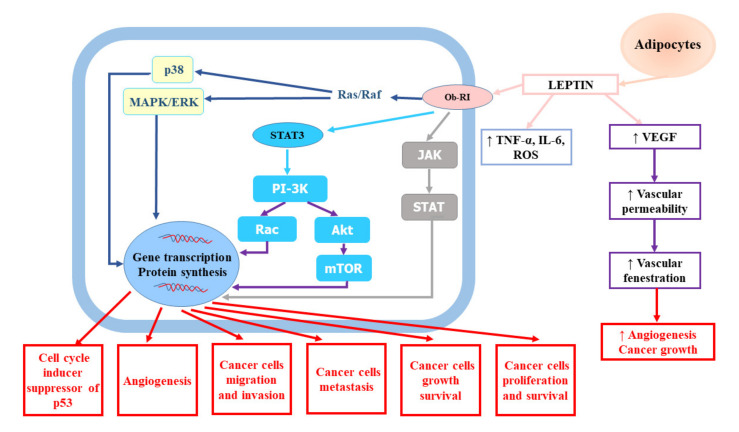
The role of leptin in renal cell cancer development and progression. Akt, protein kinase B; IL-6, interleukin-6; JAK, Janus kinase; MAPK/ERK, mitogen-activated protein kinase/extracellular-signal-regulated kinase; mTOR, mammalian target of rapamycin; Ob-RI, leptin or obesity receptor; PI-3K, phosphatidyl inositol 3-kinase; ROS, reactive oxygen species; STAT3, signal transducer and activator of transcription 3; TNF-α, tumour necrosis factor-α; VEGF, vascular endothelial growth factor.

A growing number of studies have indicated that obesity is an independent risk factor of RCC [17]. A large quantitative summary analysis performed to assess the impact of obesity on the risk of renal cell cancer both in men and women demonstrated a relationship between metabolic syndrome and RCC, which was stronger in men with higher body mass index (BMI). It also revealed that the relative risk for both sexes was 1.07 (95% confidence interval (CI): 1.05–1.09) per unit of increase in BMI (one unit corresponds to 3.1 kg for a man of average height and to 2.7 kg for a woman of average height) [22]. The meta-analysis of 24 cohort studies and nearly 9,000,000 participants confirmed weight-related increase in renal cancer risk. The risk of kidney cancer was increased by 35% in overweight participants and by 76% in obese subjects in comparison to normal weight participants, irrespective of the gender [23]. The impact of BMI on cancer risk was shown to be related to dose–response—1 kg/m^2^ increase in BMI amplified the risk by 6% in men (relative risk (RR) = 1.06, 95% CI: 1.05–1.08) and by 5% in women (RR = 1.05, 95% CI: 1.04–1.06). Hu et al. [19] showed that overweight and obesity were considerably associated with greater RCC prevalence. A large study based on data from 1,110,835 adolescent males (16–19 years) examined for fitness for military service found a substantial excess risk associated with a body mass index of greater than 27.5 kg/m^2^ compared to BMI less than 22.5 kg/m^2^ (hazard ratio (HR) = 2.43, 95% CI: 1.54–3.83, *p* < 0.0001) [24]. Authors suggested that the prevention of childhood obesity might be a promising tool for reducing the burden of renal cancer. Obesity may influence the risk of renal cancer via several mechanisms. The adipose tissue seems to be involved in the modulation of RCC risk. The adipose tissue, which is a key endocrine gland responsible for the biosynthesis and secretion of numerous hormones and cytokines (adipokines), becomes highly dysfunctional in consequence of weight gain [25]. According to studies, obesity and central fat distribution decrease serum adiponectin levels [26,27]. Adiponectin, which has been shown to play an anti-tumour role due to its anti-inflammatory and antiproliferative effects and antagonism to insulin resistance leading to the inhibition of tumour growth and angiogenesis, is mainly secreted by white adipose tissue. In vitro experiments confirmed that adiponectin hampered tumour growth through the activation of AMP-activated protein kinase (AMPK) and subsequent suppression of mammalian target of rapamycin (mTOR) pathways [28]. Moreover, Spyridopoulos et al. [29] demonstrated reverse association between serum adiponectin level and the risk of RCC. In turn, increased serum levels of leptin, which is also produced predominantly by white adipose tissue, have been found to be associated with RCC invasion and progression [30]. Numerous epidemiological studies have confirmed significant relationship between elevated leptin levels and increased risk of RCC as well as invasion, migration, and progression in RCC [31,32,33]. Some of them have indicated the association between diminished adiponectin levels and RCC tumour size [31,34]. Tumour proliferation promoted by leptin involves the activation of the extracellular signal-regulated kinases (ERK1/2) and Janus kinase/signal transducer and activator of transcription 3 (JAK/STAT3) signalling pathway, as well as the upregulation of vascular endothelial growth factor (VEGF) via hypoxia-inducible factor-1α (HIF-1α) and nuclear factor-kappa B (NF-κB), which results in apoptosis inhibition and enhanced cancer cell proliferation [30,32,33,35]. The role of leptin in renal cell cancer development and progression was presented on Figure 1.

The increased risk of RCC development in obese patients has been also ascribed to deregulation of insulin (IN) and insulin-like growth factors (IGFs) signalling since these pathways are believed to be engaged in tumorigenesis and cancer progression [36]. Obese individuals were shown to have higher oestrogen levels which may promote the carcinogenic effect of IGF [37]. Finally, obesity is associated with enhanced secretion of high amounts of pro-inflammatory cytokines, stimulating a low-grade chronic inflammatory state [25]. Growing adipose tissue also produces high levels of acute phase reactants, including serum amyloid A (SAA), C-reactive protein (CRP,) retinol-binding protein 4 (RBP4), and other proteins such as vascular cell adhesion molecule 1 (VCAM1), intercellular adhesion molecule 1 (ICAM1), and chemokine C-X-C motif ligand 10 (CXCL10) [38,39]. The presence of chronic inflammatory state can promote cancer initiation, progression, malignant conversion, invasion, and metastasis; alter host immunosurveillance; and affect local tumour microenvironment directly and distant tumour cells through the systemic effects of endocrine signals [40,41]. According to studies, inflammatory cytokines enhance the risk of recurrence following RCC surgical resection and are also associated with poor prognosis [42].

The results of numerous studies indicate that obesity increases the risk of RCC development; however, it also seems to be a favourable factor in terms of prognosis. Waalkes et al. [43] observed higher tumour grade and rate of metastasis at diagnosis in patients with lower BMI, but only those with organ-confined RCC. They indicated that the risk of cancer-related death was also significantly lower in overweight patients: median 5-year tumour-specific survival rate was 63.8% in patients with a BMI below 25 kg/m^2^, 70.9% in pre-obese subjects, 74.0% in obese (grade I) patients, and 85.6% in obese (grade ≥ II) patients (*p* < 0.001). A retrospective analysis of a database comprising 1748 patients with surgically treated RCC found significant association between high BMI and low cellular grade [44]. According to authors, the relation between high BMI and renal cancer may explain inverse association between oncological outcomes and obesity. Similar results were obtained in a study of consecutive 1975 patients with metastatic RCC who received targeted therapy (TT) in 19 centres in North America, Europe, and Asia [45]. Albiges et al. [45] observed that obese patients with both metastatic and local disease had better overall survival, after adjustment for International Metastatic Renal Cell Carcinoma Database Consortium (IMDC) prognostic factors. The overall survival of patients with high BMI was 25.6 months (95% CI: 23.2–28.6 months), while in individuals with low BMI, it was 17.1 months (95% CI: 15.5–18.5 months). Patients with high BMI also had greater time-to-treatment failure (TTF) in comparison to those with low BMI in first- and second-line therapy. A Cancer Genome Atlas (TCGA)-based analysis of biologic differences associated with different BMI groups failed to find any specific DNA alterations in clear-cell RCC in obese patients [45,46]. However, gene expression profiling revealed marked downregulation of fatty acid synthase (FASN) in obese patients in comparison to normal weight patients. Elevated FASN levels were associated in this study with worse overall survival (median, 15.0 vs. 36.8 months for high FASN and low FASN, respectively; log-rank *p* = 0.002). This finding is in agreement with the suggestion that the upregulation of FASN is a universal phenotypic alteration observed in most human malignancies, which possibly confers a survival growth advantage to tumour cells. The homeostasis of fatty acids (FAs) is controlled by sterol regulatory element-binding proteins (SREBPs) and this process has also been suggested to be essential to support cell survival and tumour growth [47,48]. Albiges et al. [45] suggested a prognostic role of the FA pathway in clear-cell renal cell carcinoma (ccRCC) since they demonstrated that FASN protein was associated with survival and with the IMDC risk groups in patients with metastatic renal cell carcinoma (mRCC) treated with TT. However, it seems that the association between obesity, FASN, and outcome is tumour-specific [45,49]. Renfro et al. [50] have suggested that inverse association between cancer prognosis and obesity, called also “obesity paradox”, could be related to a less aggressive disease subtype in obese patients. Accelerated tumour development or its higher aggressiveness may stem from dysregulation of multiple pathways, e.g., the upregulation of oestrogen and insulin levels and other growth factors secreted from adipose tissue, abnormal cholesterol metabolism, and immune system disturbances [51]. Some studies suggested that visceral adipose tissue seemed to be more harmful to health than subcutaneous adipose tissue. However, it appears that in patients with advanced RCC receiving first-line targeted therapy (i.e., sunitinib), visceral adipose tissue may play protective role. In turn, Steffens et al. [52] demonstrated that larger visceral fat area (VFA) and subcutaneous fat area (SFA) were significantly associated with better prognosis and predicted longer progression-free survival (PFS) and overall survival (OS) times. The mechanism of opposite effects of obesity on RCC risk and its prognosis has not been fully unravelled. 

Obesity is the more important risk factor associated with insulin resistance and the development of diabetes mellitus type 2 (DM2T) since the adipocytes in visceral tissue secrete substances responsible for insulin resistance, e.g., free fatty acids [8]. 

## 3. Insulin Resistance

Interactions between insulin and the IGF system appear to be of key importance in the development and progression of RCC [53]; however, the exact role of insulin and IGFs in RCC pathophysiology remains elusive. Gatica et al. [54] demonstrated significantly decreased expression of insulin receptor in patients with DM2T, insulin-resistance, and diabetic nephropathy. Insulin regulates carbohydrate and fat metabolism [55]. High serum levels of insulin have been shown to impede autophagocytosis, proteasome activity, and apoptosis, exerting anti-apoptotic and mitogenic effects [55,56]. In turn, insulin-like growth factors regulate cell growth and proliferation. The IGF family comprises IGF-1, IGF-2, their receptors (IGF-1R and IGF-2R), and six types of IGF-binding proteins (IGFBPs) [1]. The binding of ligands with IGF-1R results in the activation of tyrosine kinase signalling and the phosphorylation of insulin receptor substrate proteins (IRS), which subsequently activate the PI-3K (phosphatidyl inositol 3-kinase)/Akt (protein kinase B)/mTOR (mammalian target of rapamycin) and Ras/MAPK (mitogen-activated protein kinase) pathways [36]. These pathways have been shown to regulate apoptosis and cell proliferation and to be potentially involved in cancer development [57]. IGFs may be involved in the stimulation of mitosis and cell migration, proliferation, differentiation, and hampering of apoptosis as well as malignant transformation [58]. IGF-1 has been shown to stimulate tumour angiogenesis as a result of increasing levels of VEGF [59]. Zhang et al. [60] suggested that the stabilization of hypoxia-inducible factors and inhibition of insulin-like growth factor receptor 1 signalling resulting from von Hippel-Lindau syndrome is the essential mechanism in RCC biology. According to Johansson et al. [61], insulin resistance may lead to compensatory hyperinsulinemia resulting from enhanced insulin secretion by pancreatic β-cells to maintain normal blood glucose. It has been hypothesized that hyperinsulinemia may augment cancer cell growth and proliferation through insulin action via its receptor and the activation of IGF pathway [62]. Insulin directly and indirectly regulates liver production of IGF-1 through the upregulation of growth hormone (GH) receptors and it also increases IGF-1 bioavailability via the downregulation of IGFBP-1 and IGFBP-2 (which hinders IGF-1 actions) [63]. Suppressed serum IGFBP-1 and increased free IGF-1 have been observed in chronic or short-term hyperinsulinemia [63]. In cancer patients with insulin resistance, the elevated levels of circulating insulin accompanied frequently by insulin receptor (IR) overexpression in cancer cells may be associated with atypical stimulation of non-metabolic effects of IR, including cell proliferation, migration, and survival. Altered splicing of the IR gene resulting in the predominant expression of IR-A (which has enhanced affinity for IGFs, especially for IGF-2) further promotes the aforementioned effects [64]. 

**Figure 2:**
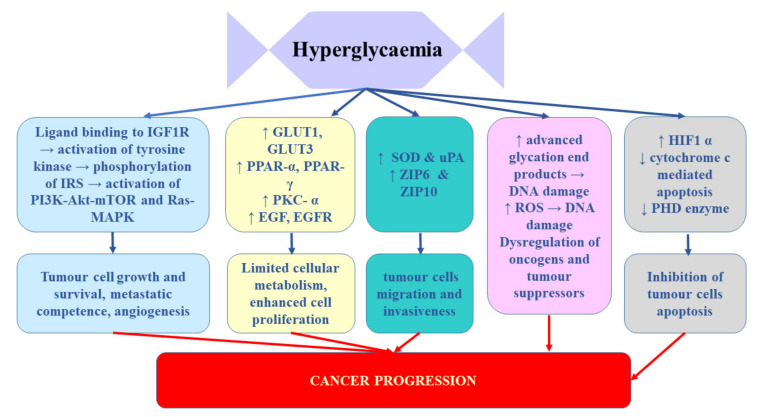
The influence of hypoglycaemia on prostate cancer development and progression. EGF, epidermal growth factor; EGFR, epidermal growth factor receptor; GLUTs, glucose transporters; HIF-1α, hypoxia-inducible factor-1α; IGF-1R, insulin-like growth factor 1 receptor; IRS, insulin receptor substrate; PHD, prolyl hydroxylase domain-containing protein; PKC-α, protein kinase C alpha; PPARs, peroxisome proliferator-activated receptors; ROS, reactive oxygen species; SOD, superoxide dismutase; uPA, urokinase-type plasminogen activator; ZIP, Zrt-/Irt-like proteins.

Takahashi [65] demonstrated that the expression of insulin receptors in RCC tissue of patients who underwent nephrectomy inversely correlated with cancer progression. It was significantly lower in patients with tumour stage pT2-4 and/or distant metastases. Moreover, enhanced IR expression strongly correlated with better disease-free and overall survival after nephrectomy. These findings suggest that hyperinsulinemia does not stimulate RCC progression [65]. Solarek et al. [36] observed downregulated IR expression in RCC cancer cells and they provided evidence that IGF signalling in RCC was predominantly mediated by circulating ligand proteins (IGF-1 and IGF-2) originating from sources other than RCC tumour itself. In turn, Rasmuson et al. [66] failed to find correlation between insulin-like growth factor-1 and IGFBP3 and tumour stage or grade. However, in their study, high serum IGF-1 levels at the time of diagnosis correlated with favourable prognosis; therefore, it seems that serum IGF-1 level may be an independent prognostic factor of renal cell carcinoma. In contrast, Johansson et al. [61] demonstrated significant association between the variables related to fasting insulin and RCC risk. In their Mendelian randomization study, one standard deviation (SD) increment in fasting insulin increased the risk of RCC by 82%. No such relationship was found for type 2 diabetes. It appears that the key mechanism linking diabetes and RCC involves chronic hyperinsulinemia related to pre-diabetic and diabetic status [67]. The impact of hypoglycaemia on prostate cancer development and progression was presented on Figure 2

## 4. Diabetes

According to studies, the prevalence of RCC in patients with diabetes mellitus ranges from 9.1% to 25.4% depending on the studied population, while the incidence of this tumour in general population accounts for approximately 3–5% [8,68,69]. DM is a metabolic disease characterized by chronic hyperglycaemia. The mechanism of DM2T development involves the presence of insulin resistance resulting from the surplus of free fatty acids and the enhanced production of pro-inflammatory cytokines followed by overproduction of insulin by the pancreas as a compensatory mechanism (initially) to insulin resistance and gradual degeneration of pancreatic β-cells [8]. In the course of insulin resistance, adipocytes may release pro-inflammatory cytokines, which may induce DNA damage resulting finally in carcinogenesis [1]. This thesis was confirmed in a study based on a microarray profile analysis of human kidney from diabetes, renal cell carcinoma, and renal cell carcinoma with diabetes, which revealed numerous DNA modifications in patients from the RCC+diabetes group [70]. In their study, insulin receptor was not only highly expressed, but it also had gains in copy number in patients with diabetes and RCC and in patients with diabetes only. The authors suggested that the analysis of IR copies might be useful as a biomarker allowing the prediction of RCC development in diabetic patients. Since renal cancer is predominantly a metabolic disease, it seems that mutations related to disorders in oxygen, iron, nutrient, and/or energy sensing provide the foundation for the development of cancers and they are indeed frequently observed in renal cancer syndromes [71].

The most commonly mentioned carcinogenic factor observed in the course of diabetes mellitus involves hyperglycaemia, as according to Warburg hypothesis, tumour cells require large amounts of glucose since they use aerobic glycolysis to provide energy and biomolecules regardless of the availability of oxygen [8]. Prolonged hyperglycaemic condition has been observed to result in severe diabetic condition through the damage of pancreatic β-cells, damage of vascular endothelial cells, and induction of insulin resistance. The resulting β-cell dysfunction stimulates diminished insulin synthesis and secretion, further aggravating the associated hyperglycaemia. In pathological conditions of hyperinsulinemia, insulin can trigger the proliferation pathway activated by IGF-1 in kidney cancer cells lines. Moreover, IR and IGF-1R were found to promote cellular proliferation and induce metastasis in experimental models [72]. Hyperglycaemia was suggested to stimulate cancer cell proliferation by rising the levels of protein kinase C (PKC) and peroxisome proliferator-activated receptors (PPARs), which in turn can hasten cellular metabolism and promote proliferation [73]. Moreover, the presence of DM is associated with the hyperactivation of protein kinase B (Akt)/mTOR regulating most of the cellular pathways, including those involved in renal carcinogenesis. This thesis was supported by a study in which elevated levels of phosphorylated p70S6K (its phosphorylation is caused by activated mTOR kinase) were detected in patients with both DM and RCC and in patients with only DM [74]. Finally, hyperinsulinemia and the insulin-like growth factor family have been suspected to participate in diabetes-related RCC risk. Insulin, as it was described in the previous section, promotes the synthesis and activity of IGF-1 as well as mitosis and cell proliferation and thus, it exerts tumour growth-stimulating effects.

Some studies indicated that the administration of metformin, which is a commonly used anti-diabetic drug, inhibited the growth of renal tumour cells via the stimulation of apoptosis, downregulation of cyclin D1 expression, and G0/G1 cell cycle arrest, as well as reduced cancer mortality in patients with DM2T [75,76,77]. Moreover, metformin was shown to stimulate AMPK and to inhibit mTOR that plays a role in cell proliferation, differentiation, growth, and survival [76]. If the therapeutic efficiency of metformin is confirmed, it will mean that DM may play an important role in the pathophysiology of RCC [78]. 

Numerous studies have indicated the relationship between diabetes mellitus and cancers suggesting that some of the DM risk factors can increase an individual’s susceptibility to develop a tumour [1]. A large prospective study demonstrated a statistically significant greater risk of developing RCC in diabetic women in comparison to those without type 2 diabetes (multivariable HR = 1.53; 95% CI: 1.14–2.04) and this association was independent of obesity, hypertension, and smoking and was the strongest for non-clear-cell RCC [79]. A meta-analysis of 24 studies found considerable relation between diabetes and increased risk of kidney cancer (RR = 1.40, 95% CI: 1.16–1.69). Again, a slightly stronger positive correlation was observed in women (RR = 1.47, 95% CI: 1.18–1.83) than in men (RR = 1.28, 95% CI: 1.10–1.48) [80]. The results of these studies may suggest the existence of a different mechanism underlying the impact of type 2 diabetes on RCC risk in women and men, since in the general population, the risk of RCC is greater in men than in women. The impact of female hormones (including oral contraceptives and postmenopausal hormone use) on RCC risk has been analysed; however, the results of studies are conflicting [81,82].

According to Lee et al. [78], diabetes may not significantly affect the aggressiveness and/or progression of RCC. In turn, epidemiological studies imply the relationship between diabetes and enhanced morbidity and mortality of RCC; however, the results of studies are sometimes conflicting. In patients with surgically treated clear-cell renal cell carcinoma, the 5-year overall survival was significantly worse in those with diabetes (65% vs. 74%, *p* < 0.001) [20]. Moreover, a multivariable analysis revealed that diabetes mellitus independently predicted cancer-specific mortality (HR = 1.55, 95% CI: 1.08–2.21, *p* = 0.02) and all-cause mortality (HR = 1.32, 95% CI: 1.06–1.64, *p* = 0.01).

**Figure 3:**
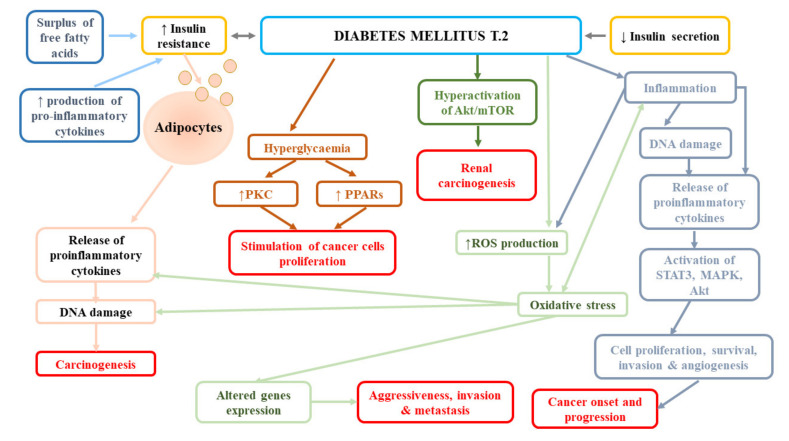
Pathomechanisms related to cancer development and progression in diabetes mellitus. Akt, protein kinase B; MAPK, mitogen-activated protein kinase; PKC, protein kinase C; PPARs, peroxisome proliferator-activated receptors; ROS, reactive oxygen species; STAT3, signal transducer and activator of transcription 3.

In contrast, the follow-up of participants from the Nurses’ Health Study (NHS) and the Health Professionals Follow-Up Study (HPFS) failed to find the effect of diabetes on the incidence of fatal RCC in both women (HR = 1.35, 95% CI: 0.72–2.55) and women (HR = 1.23, 95% CI: 0.54–2.76) [79]. Similarly, a large meta-analysis reported no association between diabetes and mortality of kidney cancer (RR = 1.12, 95% CI: 0.99–1.20) [80]. In turn, Lee et al. [78] observed significantly lower overall survival, cancer-specific survival (CSS), and non-cancer-related survival in patients with diabetes mellitus and renal cancer in comparison to patients without diabetes. The authors suggested that it was not the presence/absence of diabetes that affected RCC-related outcomes, but the time from diagnosis of DM or degree of long-term glycaemic control. Moreover, they reported that the presence of DM before surgical treatment was a strong predictor of overall survival and non-cancer-specific survival, but not cancer-specific survival in patients with localized clear-cell RCC, following the adjustment for numerous pre-operative variables and known prognostic factors. The impact of DM presence on overall and non-cancer-specific survival may be associated with the fact that this disease increases the risk of cardiovascular mortality. Indeed, in the abovementioned study, most of non-RCC-related mortality was due to cerebrovascular and heart-related conditions [78]. In a subsequent study performed by Lee et al. [83], diabetes was an independent predictor of disease progression (HR = 1.766, *p* = 0.002), cancer-specific mortality (HR = 2.266, *p* = 0.001), and all-cause mortality (odds ratio [OR] = 1.825, *p* = 0.001), while elevated pre-operative HbA1c was shown to predict post-operative disease progression (HR = 2.221, *p* = 0.023) A long-term retrospective single-centre study demonstrated a higher risk of all-cause mortality in patients with RCC and pre-existing DM2T at the time of diagnosis compared to individuals without diabetes [84]. However, they noticed the impact of DM on cancer-specific survival; the estimated CSS rates in patients with DM2T vs. non-DM2T were as follows: at 1 year, 63.4% vs. 76.7%; at 3 years, 30.4% vs. 56.6%; and at 5 years, 16.3% vs. 48.6% (*p* = 0.001). Moreover, Yuk et al. [12] found significantly shorter progression-free, cancer-specific, and overall survival of patients with pre-operative diabetes mellitus than non-DM ones (all *p* < 0.05), as well as worse oncological outcomes in diabetic individuals with poor glycaemic control compared to those with good glycaemic control. Finally, Fukushima et al. [85] reported the relationship between DM and the rate of recurrences following surgery for non-metastatic renal cell carcinoma (HR = 2.43, *p* = 0.003), especially in obese patients (HR = 4.07, *p* = 0.010). Obesity altered the impact of diabetes mellitus on recurrence with a trend (*p*-interaction = 0.086). The 5-year recurrence-free survival rate in obese patients with DM was 75.3%, while in obese patients without DM, it was 91.9% (*p* < 0.001). Figure 3 presents pathomechanisms related to cancer development and progression in diabetes mellitus.

## 5. Dyslipidemia

Lipid disorders have been suggested to be involved in the pathomechanisms of various cancers, including oesophageal, colon, rectal, and renal cancers [86,87]. According to numerous studies, clear-cell renal cancer is also characterized by sterol storage in tumour cells resulting in alterations in lipid metabolism and consequently, in the formation and progression of RCC [1,88]. However, the exact mechanisms are not fully understood as the results of studies are sometimes conflicting. Some studies have demonstrated decreased cancer incidence and mortality in patients with high baseline levels of total cholesterol (TC), while other have indicated an inverse relationship or the existence of a U-shaped association with TC levels [89,90,91,92,93,94].

A prospective cohort study (Swedish AMORIS study) demonstrated a consistent relation only between triglyceride (TG) levels and kidney cancer risk [18]. A 1:2-matched case–control study, in which renal cell carcinoma patients were matched (in terms of age and gender) to doubled number of non-renal-cell-carcinoma residents, found a relationship between low high-density lipoprotein (HDL) levels and greater risk of renal cell carcinoma (HR = 1.646, 95% CI: 1.187–2.282) as well as between high levels of TC (HR= 0.537, 95% CI: 0.374–0.772) and high concentration of low-density lipoprotein (LDL) (HR = 0.649, 95% CI: 0.442–0.971) and diminished risk of renal cell carcinoma, after adjusting for obesity, smoking, hypertension, and diabetes [95]. The increase in low-density lipoprotein cholesterol (LDL-C) level by 1 mmol/L was associated with 25.5% reduction in renal cell cancer hazard. On the basis of obtained results, Zhang et al. [95] suggested that the impact of HDL on carcinogenesis might involve its effects on cytokine production, anti-oxidative properties, the regulation of cell cycle entry via a mitogen activated protein kinase-dependent pathway, and apoptosis, but it was also plausible that other factors affected both HDL cholesterol levels and the risk of cancer development [96,97,98]. Similarly, a Mendelian randomization study including participants from the Copenhagen City Heart Study (CCHS) and Copenhagen General Population Study revealed that low plasma levels of LDL cholesterol (<87 mg/dL) were strongly associated with 43% increased risk of cancer (including kidney cancer) in comparison to individuals with LDL >158 mg/dL (95% CI: 15%–79% increase) [99]. Their finding did not apply to patients with genetically reduced LDL cholesterol levels, which implies that low LDL cholesterol levels per se do not cause cancer. In contrast, a retrospective case–control study of patients with newly diagnosed sporadic RCC [88] reported an upward trend for the occurrence of advanced RCC with rising serum LDL levels. Elevated levels of LDL also frequently co-existed with the presence of clear-cell RCC. In turn, Ahn et al. [100] demonstrated a marginally significant association between high serum concentrations of total cholesterol and a reduced risk of kidney cancer. Another European cohort study reported the relationship between triglyceride and RCC hazard only in men, but not in women [10]. In a retrospective study investigating the association between metabolic factors and the histopathological characteristics of renal cell carcinoma, patients in the high-grade group were observed to have lower HDL cholesterol in comparison to the low-grade group (*p* = 0.015) [101]. Lower HDL cholesterol and total cholesterol level were found in those with more advanced cancer than those with localized disease (*p* = 0.006 and *p* = 0.005, respectively). Lower concentrations of LDL cholesterol correlated with the presence of larger tumours (*p* = 0.030). Finally, logistic analyses performed in this study indicated associations between total cholesterol and tumour stage (OR = 0.660, 95%CI: 0.492–0.884, *p* = 0.005), HDL cholesterol level and tumour grade (OR = 0.293, 95%CI: 0.108–0.797, *p* = 0.016), and stage (OR = 0.204, 95%CI: 0.065–0.635, *p* = 0.006), as well as LDL levels and tumour diameter (OR = 0.756, 95%CI: 0.586–0.975, *p* = 0.031) [101].

**Figure 4:**
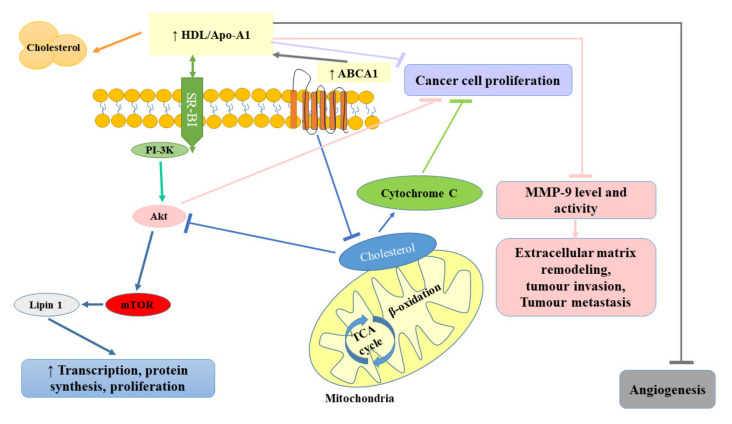
Protective role of high-density lipoprotein (HDL) in cancer development. ABCA1, ATP-binding cassette transporter; Akt, protein kinase B; Apo-A1, apolipoprotein A-I; HDL, high-density lipoprotein; MMP-9, matrix metallopeptidase-9; mTOR, mammalian target of rapamycin; PI-3K, phosphatidyl inositol 3-kinase; SR-BI, scavenger receptor class B type 1; TCA, trichloroacetic acid.

Some studies indicated that the administration of statins (inhibitors of 3-hydroxy-3-methylglutaryl coenzyme A reductase) used in the treatment of lipid disorders seemed to exert protective effects against the development of RCC [1,102]. Horiguchi et al. [102] in their in vitro experimental model assessed the therapeutic efficacy of fluvastatin against renal cancer growth, invasion, angiogenesis, and pulmonary metastasis. They observed that fluvastatin even at relatively low concentrations (0.1 and 1 μmol/L) diminished Renca cell proliferation, with up to 70% inhibition at 10 μmol/L. The observed inhibitory effect was suggested to be related to the induction of apoptosis. Doses of fluvastatin recommended for the treatment of hypercholesterolemia were also shown to exert an antiproliferative effect by blocking the transition of G1–S in the cell cycle as a result of the increase in p21WAF/CIP1 (cyclin-dependent kinase inhibitor) and to significantly hamper renal cancer cell invasion and angiogenesis, resulting in reduced pulmonary metastasis [102]. Since the treatment with oral fluvastatin is well-tolerated and cost-effective, it seems that it could be used as an effective treatment for preventing the invasion and pulmonary metastasis of renal cancer cells. Figure 4 summarizes the protective role of HDL in cancer development.

Table 1 presents the summary of most important studies included in this review.

It is worth mentioning that cancer may cause protein-energy wasting syndrome, which affects metabolic factors evaluated in this review; therefore, some of the disturbances described here could be related to the disease. Metabolic impairment contributes to the clinical deterioration seen in advanced cancer patients, including weight loss, skeletal muscle wasting, as well as the atrophy of the adipose tissue [103]. Cancer-associated cachexia (CAC) progresses rapidly resulting in irreversible deterioration of health and survival; however, its appearance seems to be mostly unpredictable and the severity of CAC is frequently not related to tumour size or stage. Metabolic dysfunction in cancer results from the deregulation of carbohydrate and lipid metabolism. Tumour tissue takes up glucose, therefore the decreased levels of glucose observed in such patients can be ascribed to cancer development. Reduced glucose tolerance observed in cancer patients could be ascribed to enhanced hepatic glucose production or its diminished peripheral utilization [103]. Moreover, in severely sick patients with cancers, a paradoxical rise in serum lipid levels was observed despite the loss of body weight. According to some studies, hypertriglyceridemia could be the result of the inhibition of lipoprotein lipase (LPL) [104].

## 6. Conclusions

The results or numerous studies suggests that metabolic factors, especially diabetes mellitus, obesity, and serum lipid profile, are closely associated with the risk of renal cell carcinoma, as well as its histopathological characteristics and survival. Worse outcomes seem to be the result of the presence of metabolic factors per se. It appears that obesity increases the risk of RCC development; however, it may also be a favourable factor in terms of prognosis. Obesity is closely related to insulin resistance and the development of diabetes mellitus type 2 since the adipocytes in visceral tissue secrete substances responsible for insulin resistance, e.g., free fatty acids. Interactions between insulin and the IGF system appear to be of key importance in the development and progression of RCC; however, the exact role of insulin and IGFs in RCC pathophysiology remains elusive. Studies indicated that diabetes increased the risk of RCC, but it might not alter cancer-related survival. The risk associated with lipid profile is most mysterious, as numerous studies provided conflicting results. Despite the fact that large studies unravelling pathomechanisms involved in cancer growth are required to finally establish the impact of metabolic factors on the development, progression, and prognosis of renal cancers, it seems that the monitoring of health conditions, such as diabetes, low BMI, and lipid disorders is of high importance in clear-cell type RCC. There are still numerous questions awaiting clarification. More and more sophisticated techniques of molecular biology should enable the identification of key metabolic enzymes and pathways involved in cancer development and progression. Metabolic profiling of samples from cancer and non-cancer patients could provide relevant information on whether there exists a specific, characteristic metabolic profile of tumour cells that is different from that of non-transformed proliferating cells. Perhaps future studies will provide the information if metabolic particularities of cancer cells can be exploited without affecting normal tissues. Moreover, it is of interest how the diet or DM treatment influences cancer development and therapy.

## Figures and Tables

**Table 1:** The summary of most important studies included in this review.

Factor	Type of Study	Study Group	Most Important Results	Reference
**Obesity, leptin, and adiponectin levels**	Quantitative summary analysis	14 studies on men and women	➢ Summary relative risk estimate was 1.07 (95% confidence interval (CI): 1.05–1.09) per unit of increase in body mass index (BMI) corresponding to 3 kg body weight increase for a subject of average height). ➢ No evidence of effect modification by sex. **Conclusion**: Increased BMI is equally strongly associated with an increased risk of renal cell cancer among men and women.	[22]
	Meta-analysis in accordance with PRISMA guideline	24 cohort studies with 8,953,478 participants	➢ Pooled relative risk (RR) of kidney cancer was 1.35 (1.27–1.43) in overweight and 1.76 (1.61–1.91) in obese participants compared to those with normal weight. ➢ Increased kidney cancer risk of 1.06 (1.05–1.06) for each 1 kg/m^2^ increase in BMI (risk increased by 6% in men (RR = 1.06, 95%CI: 1.05–1.08) in men and 5% in women (RR = 1.05, 95% CI: 1.04–1.06). ➢ **Conclusion**: The overall and dose-response meta-analysis suggested that overweight/obesity increases the risk of kidney cancer both in men and women.	[23]
	Nationwide, population-based cohort study, also based on Israel National Cancer Registry	1,110,835 males, aged 16–19 years, examined for fitness for military service; 274 of them developed renal cancer	➢ Substantial excess risk related to body mass index of greater than 27.5 kg/m^2^ compared with less than 22.5 kg/m^2^ (hazard ratio (HR) = 2.43, 95% CI: 1.54–3.83, *p* < 0.0001). ➢ Asian or African origin was protective compared with European origin (African origin: HR = 0.67, 95% CI: 0.49–0.92). **Conclusion**: Overweight in late adolescence is a substantial risk factor for renal cell carcinoma. European origin is independently associated with excess risk.	[24]
	Case–control study	70 patients with histologically confirmed renal cell carcinoma (RCC) and 280 healthy controls matched by gender, age, and county of residence	➢ Serum adiponectin levels were statistically, significantly, and inversely associated with RCC when compared with controls (odds ration [OR] = 0.76, *p* = 0.05). **Conclusion**: Low adiponectin levels are significantly associated with RCC and may mediate the effect of central or visceral adiposity on the pathogenesis of RCC.	[29]
	Case–control study	Caucasians (581 cases, 558 controls) and African Americans (187 cases, 359 controls)	➢ In Caucasians, the ORs for RCC comparing the highest (Q4) to the lowest (Q1) sex-specific quartile of leptin were 3.2 (95% CI: 1.9–5.2) for males and 4.7 (95% CI: 2.6–8.6) for females. ➢ Serum leptin was not significantly associated with RCC among African American males or females. ➢ Higher adiponectin was associated with RCC risk among African American males (Q4 vs. Q1: OR = 2.3, 95% CI: 1.1–4.6) and females (OR = 2.1, 95% CI: 1.2–6.7). ➢ Adiponectin was not significantly associated with RCC risk among Caucasian males and females. **Conclusion**: Leptin and adiponectin concentrations are associated with the risk of RCC, which may differ by race.	[31]
	Case–control study	1338 clear-cell RCC patients with complete information about their BMI	➢ Lower BMI was significantly associated with higher age, tumour grade, and the rate of metastasis at diagnosis. ➢ Significantly lower risk of cancer-related death in overweight patients (median 5-year tumour-specific survival rate: 70.9% (pre-obese), 74.0% (obese grade I), and 85.6% (obese grade ≥ II) compared with 63.8% for patients with BMI below 25 (*p* < 0.001). ➢ Overweight is an independent prognostic marker of improved cancer-specific survival in patients with organ-confined, but not advanced, RCC.	[43]
	International, multi-institutional retrospective review	1748 patients with median BMI of 28 who underwent surgery for clinically localized renal masses	➢ Increased distribution of low-grade RCC with increasing BMI (*p* < 0.05). ➢ In a multivariable model (including age, sex, and tumour size), higher BMI groups had lower odds of presenting a high Fuhrman grade. **Conclusions**: Higher BMI is associated with a lower grade of RCC in clinically localized renal masses.	[44]
	Case–control study	1975 patients from the International Metastatic Renal Cell Carcinoma Database Consortium (IMDC), external validation cohort of 4657 patients.	➢ High BMI was associated with improved overall survival (OS; adjusted hazard ratio = 0.83, 95% CI: 0.74–0.93). ➢ Fatty acid synthase (FASN) immunohistochemistry positivity was more frequently detected in IMDC poor (48%) and intermediate (34%) risk groups than in the favourable risk group (17%; *p*-trend = 0.015). ➢ FASN protein levels were associated with survival in patients with metastatic RCC (mRCC). **Conclusions**: High BMI is a prognostic factor for improved survival and progression-free survival in patients with metastatic RCC treated with targeted therapy. FASN pathway is involved in RCC risk.	[45]
	Case–control study	116 patients with metastatic RCC receiving anti-angiogenic agents (sunitinib, sorafenib, axitinib, bevacizumab)	**Conclusions**: Higher than average visceral fat area (VFA) and subcutaneous fat area (SFA) levels were significant predictors of longer progression-free and overall survival times.	[52]
**Insulin resistance**	Case–control study	Patients with RCC who underwent nephrectomy	➢ Significantly lower insulin receptor (IR) expression level in RCC tissue in patients with tumour stage pT2-4 and/or distant metastases. ➢ High IR expression level was significantly associated with better disease-free and overall survival after nephrectomy. ➢ IR expression in RCC tissue was inversely associated with cancer progression. **Conclusions**: Hyperinsulinemia does not promote RCC progression. Decreased IR expression in high-stage RCC tumours with poor prognosis may be the result of downregulation induced by the host’s hyperinsulinemia.	[65]
	Case–control study	256 consecutive patients with renal cell carcinoma	➢ Lack of correlation between insulin-like growth factor-1, IGFBP-3, and tumour stage or grade. ➢ Inverse correlation between leptin and pre-albumin and tumour stage and grade. ➢ Tumour stage and serum IGF-1 levels are independent prognostic factors. **Conclusions**: Serum IGF-1 at diagnosis is related to prognosis in renal cell carcinoma.	[67]
	Genome-wide association study (GWAS)	10,784 RCC patients and 20,406 control participants	➢ Higher body mass index increased the risk of RCC (odds ratios for a standard deviation [ORSD] = 1.56, 95% CI: 1.44–1.70). ➢ Higher fasting insulin (ORSD = 1.82, 95% CI: 1.30–2.55) and diastolic blood pressure (DBP; ORSD = 1.28, 95% CI: 1.11–1.47) increased the risk for RCC. ➢ **Conclusions**: Insulin plays an etiological role in RCC, obesity, and DBP influence RCC risk	[61]
**Diabetes**	Screening of whole human DNA genome	Healthy control, patients with diabetes or renal cell carcinoma (RCC) or RCC + diabetes	➢ Majority of DNA alterations were in patients from RCC + diabetes group. ➢ Insulin receptor was highly expressed and had gains in copy number in RCC + diabetes and diabetes only groups. **Conclusions**: The changes in insulin receptor (INSR) copy number may be used as a biomarker for predicting RCC development in diabetic patients.	[70]
	Case–control study	117,570 women from the Nurses’ Health Study (NHS) including 418 RCC case subjects and 48,866 men from the Health Professionals Follow-Up Study (HPFS) including 302 RCC case subjects	➢ Women with type 2 diabetes had a significantly increased risk of RCC compared with women without type 2 diabetes (multivariable HR = 1.53, 95% CI: 1.14–2.04), with some evidence that the association was stronger for ≤5 (HR = 2.15, 95% CI: 1.44–3.23) than >5 (HR = 1.22, 95% CI: 0.84–1.78) years’ duration of type 2 diabetes (*p*-difference = 0.03). **Conclusions**: Type 2 diabetes was independently associated with a greater risk of RCC in women but not in men.	[79]
	Meta-analysis	24 studies comprising patients with diabetes and RCC	➢ Significant association between diabetes and increased risk of kidney cancer (RR = 1.40, 95% CI: 1.16–1.69). ➢ A slightly stronger positive relation in women (RR = 1.47, 95% CI: 1.18–1.83) than in men (RR = 1.28, 95% CI: 1.10–1.48). ➢ No association between diabetes and mortality of kidney cancer (RR = 1.12, 95% CI: 0.99–1.20). **Conclusions**: Diabetes mellitus may increase the risk of kidney cancer in both women and men.	[80]
	Review of patients’ data	950 patients who received radical or partial nephrectomy for localized clear-cell renal cell carcinoma	➢ Non-diabetic patients had superior survival rates (cancer-specific, overall, and non-cancer-related survival) than diabetics (*p* = 0.012, *p* < 0.001, and *p* < 0.001, respectively). ➢ Diabetes mellitus was shown to be an independent predictor of overall survival (*p* = 0.022) and non-cancer-related survival (*p* = 0.034). **Conclusions**: Diabetes mellitus may not be directly associated with disease-specific outcome in patients who receive surgical management for localized renal cell carcinoma.	[78]
	A propensity score matching study	3075 consecutive patients treated with radical or partial nephrectomy for non-metastatic renal cell carcinoma	➢ Patients with diabetes had worse prognosis in terms of progression-free, overall and cancer-specific survival (each *p* < 0.001) (before matching). ➢ In matched cohorts, patients with diabetes showed progression-free (*p* = 0.001), cancer-specific (*p* < 0.001), and overall survival (*p* < 0.001) inferior to that of patients without diabetes. ➢ Multivariate analyses: Diabetes was an independent predictor of disease progression (HR = 1.766, *p* = 0.002), all-cause mortality (OR = 1.825, *p* = 0.001), and cancer-specific mortality (HR = 2.266, *p* = 0.001). **Conclusions**: Diabetes mellitus (DM) is an independent predictor of cancer-specific and overall survival in patients who undergo surgery for RCC. Poor glycaemic control is associated with a higher risk of progression.	[83]
	Long-term retrospective study	924 patients treated by radical or partial nephrectomy for sporadic, unilateral RCC	➢ The estimated cancer-specific survival (CSS) rates at 1, 3, and 5 years in diabetes mellitus type 2 (DM2T) vs. non-DM2T patients: 63.4% vs. 76.7%, 30.4% vs. 56.6%, and 16.3% vs. 48.6%, respectively (*p* = 0.001). ➢ Mean progression-free survival (PFS) was significantly lower (31.5 vs. 96.3 months, *p* < 0.0001) in the DM2T group. ➢ DM2T was an independent adverse prognostic factor for OS (HR = 3.44, 95% CI: 2.40–4.92), CSS (HR = 6.39, 95% CI: 3.78–10.79), and PFS (HR = 4.71, 95% CI: 3.11–7.15). **Conclusions**: Patients with RCC and pre-existing DM2T have a shorter OS, increased risk of recurrence, and higher risk for kidney cancer mortality than those without diabetes.	[84]
	Retrospective study	543 patients with non-metastatic renal cell carcinoma who underwent radical or partial nephrectomy	➢ Diabetes mellitus was an independent predictor of RCC recurrence (HR = 2.43, *p* = 0.003). ➢ Obesity modified the effect of diabetes mellitus on recurrence with a trend (*p*-interaction = 0.086). **Conclusions**: Diabetes mellitus is a predictor of recurrence following surgery for non-metastatic renal cell carcinoma, especially in obese patients.	[85]
**Dyslipidemia**	Large prospective cohort study	542,924 persons from the Swedish Apolipoprotein Mortality Risk study (including 958 persons who developed kidney cancer)	➢ Triglycerides (TGs) significant correlated with kidney cancer risk (HR = 1.25, 95% CI: 0.99–1.60; HR = 1.29, 95% CI: 1.01–1.66; and HR = 1.66, 95% CI: 1.30–2.13) for the 2nd, 3rd, and 4th quartile, respectively, compared to the 1st, with a *p*-value of <0.001 for trend.	[18]
	1:2 Matched case–control study	248 in-patients with a primary diagnosis of RCC; controls sampled from a community survey database	➢ Association between elevated serum cholesterol (*p* < 0.001), low-density lipoprotein (LDL) cholesterol (*p* < 0.001), and high-density lipoprotein (HDL) cholesterol (*p* = 0.003) and decreased hazard of RCC (adjustment for obesity, smoke, hypertension, and diabetes). ➢ Increase in low-density lipoprotein cholesterol (LDL-C) level by 1 mmol/L was associated with 25.5% reduction in renal cell cancer hazard. **Conclusions**: Abnormal lipid profile influences the risk of renal cell carcinoma.	[95]
	Mendelian randomization study	10,613 participants in the Copenhagen City Heart Study (CCHS) and 59,566 participants in the Copenhagen General Population Study, 6816 of whom had developed cancer	➢ LDL < 87 mg/dL was associated with a 43% increase (95% CI: 15%–79% increase) in the risk of cancer. ➢ The polymorphisms were associated with up to a 38% reduction (95% CI: 36%–41% reduction) in LDL cholesterol levels, but not with increased risk of cancer. **Conclusions**: Low plasma LDL cholesterol levels were robustly associated with an increased risk of cancer, but genetically decreased LDL cholesterol was not. This finding suggests that low LDL cholesterol levels per se do not cause cancer.	[99]
	Retrospective study	382 consecutive RCC patients who underwent radical or partial nephrectomy	➢ Patients in the high-grade cancer group had lower HDL-cholesterol level than those in the low-grade group (*p* = 0.015). ➢ Total cholesterol levels were lower in advanced disease than in localized disease (*p* = 0.005). ➢ LDL-cholesterol was lower in larger tumours (*p* = 0.030). **Conclusions**: Metabolic factors, especially obesity and serum lipid profile, are closely related with the histopathological characteristics of renal cell carcinoma.	[88]
